# The role of Smad7 in oral mucositis

**DOI:** 10.1007/s13238-014-0130-4

**Published:** 2015-01-08

**Authors:** Li Bian, Gangwen Han, Carolyn W. Zhao, Pamela J. Garl, Xiao-Jing Wang

**Affiliations:** 1 Department of Pathology, The First Affiliated Hospital of Kunming Medical University, Kunming, 650032 China; 2 Department of Pathology, University of Colorado Anschutz Medical Campus, Aurora, CO 80203 USA; 3 Department of Dermatology, The Second Hospital of Shandong University, Jinan, 250033 China

**Keywords:** Smad7, oral mucositis, cancer, TGFβ, NF-κB

## Abstract

Oral mucositis, a severe oral ulceration, is a common toxic effect of radio- or chemoradio-therapy and a limiting factor to using the maximum dose of radiation for effective cancer treatment. Among cancer patients, at least 40% and up to 70%, of individuals treated with standard chemotherapy regimens or upper-body radiation, develop oral mucositis. To date, there is no FDA approved drug to treat oral mucositis in cancer patients. The key challenges for oral mucositis treatment are to repair and protect ulcerated oral mucosa without promoting cancer cell growth. Oral mucositis is the result of complex, multifaceted pathobiology, involving a series of signaling pathways and a chain of interactions between the epithelium and submucosa. Among those pathways and interactions, the activation of nuclear factor-kappa B (NF-κB) is critical to the inflammation process of oral mucositis. We recently found that activation of TGFβ (transforming growth factor β) signaling is associated with the development of oral mucositis. Smad7, the negative regulator of TGFβ signaling, inhibits both NF-κB and TGFβ activation and thus plays a pivotal role in the prevention and treatment of oral mucositis by attenuating growth inhibition, apoptosis, and inflammation while promoting epithelial migration. The major objective of this review is to evaluate the known functions of Smad7, with a particular focus on its molecular mechanisms and its function in blocking multiple pathological processes in oral mucositis.

## ORAL MUCOSITIS FREQUENCY

Oral mucositis, a severe oral ulceration, is a common toxic effect of radiation for bone marrow transplant and upper body radiotherapy or chemo-radiotherapy for all cancer types (Sonis, 2010; Vagliano et al., 2011). Severe oral mucositis is extremely painful and impairs food/liquid intake, and is rated by patients as the most debilitating side effect of cancer therapy (Bellm et al., 2000). At least 40%, and up to 70%, of individuals treated with standard chemotherapy regimens or upper-body radiation develop oral mucositis (Scully et al., 2003). Among them, over 80% of oral cancer patients (the 6th most common cancer worldwide) are treated with radiation and at least 70% develop severe oral mucositis (Trotti et al., 2003; Vera-Llonch et al., 2006). The use of intensity modulated radiotherapy (IMRT) spares more normal tissue and lessens chronic side effects, but does not alleviate the acute toxicity seen in oral mucosa (Khuntia D et al., 2008) and certain patients are at a high risk of oral mucositis regardless of treatment regimen (Scully et al., 2006). Further, many high risk patients experience amplified acute oral mucositis when receiving radiotherapy combined with targeted therapies (Sonis, 2013). In the US, there are at least one million oral mucositis patients every year. Costs to care for oral mucositis can be up to $6,000 per patient per chemo- or radiation cycle for narcotic therapy, antibiotics, anemia, liquid diet, additional office visits, and nursing staff costs. For more severe oral mucositis cases, when a feeding tube and additional hospitalization are needed, the cost of associated care can be up to $25,000/cycle. Because there is no effective treatment, oral mucositis lesions are often serious enough to delay, reduce or halt cancer therapy. Palifermin, a human keratinocyte growth factor (KGF) recombinant protein, is the only FDA approved targeted therapy for preventing oral mucositis in bone-marrow transplant patients (4% of the at-risk population), but it has no effect on existing mucositis (Sonis, 2010). Palifermin clinical trials in head and neck cancer patients showed only modest preventive effects on severe oral mucositis incidence (Henke et al., 2011; Le et al., 2011). The incidence and severity of mucositis depends on multiple factors, including chemotherapeutic agents and doses, radiation regimen, patient age, gender, nutritional status, oral microbiota, salivary secretory function, and genetic variation (Barasch and Peterson, 2003; Sonis, 2009).

## PATHOLOGICAL PROCESSES OF ORAL MUCOSITIS

Oral mucositis is characterized by an infiltration of excessive inflammatory cells, epithelial ablation, and ulcer development (Scully et al., 2006; Wu et al., 2010). Oral mucositis consists of five overlapping stages: initiation, damage response, signal amplification, ulceration, and healing. At the initiation stage, radiotherapy or chemotherapy injures the tissue through DNA strand breaks, cell death in the basal and suprabasal layers, and halting epithelial proliferation. Additionally, the injured tissue produces reactive oxygen species (ROS), which in turn induce oxidative stress-associated DNA damage. Apart from the epithelial injury, radiotherapy and chemotherapy also exacerbate the mucosal damage by effecting the underlying submucosa, including endothelia, fibroblasts, and leukocytes (Treister and Sonis; Denham and Hauer-Jensen, 2002; Sonis, 2004a; Lalla and Peterson, 2006). During the damage response stage, submucosal cells expressing early response genes, such as c-jun, c-fos, and Erg-1, join in the intricate series of biological cascades, and several signal transduction pathways are activated. Among them, NF-κB is one of the most significantly activated pathways, which upregulates many genes to elicit a wide range of tissue responses and affect the process of oral mucositis (Sonis, 2002; Sonis, 2004a). At the signaling amplification stage, pro-inflammatory cytokines such as NF-κB, tumor-necrosis factor-α (TNF-α), interleukin-1β (IL-1β), interleukin-6 (IL-6), ceramide, and matrix metalloproteinases (MMPs), are overproduced. Mucosal damage triggers the initial release of these factors, leading to the positive feedback loop that amplifies mucosal damage (Sonis, 2002; Sonis, 2004a). At the ulceration stage, the integrity of the epithelium disintegrates and ulceration occurs with a massive infiltration of leukocytes in response to both tissue injury and secondary microbial infection. (Treister and Sonis; Sonis, 2004a). Subsequently, tissue begins to heal. In the healing stage, the epithelium is re-epithelialized by migrating keratinocytes followed by keratinocyte proliferation to restore normal epithelial layers that are remodeled by differentiation. Inflammation is reduced, and stromal fibroblasts as well as vessels are remodeled (Treister and Sonis; Sonis, 2004a).

## ORAL MUCOSITIS CARE AND EXPERIMENTAL THERAPEUTICS

Due to the complex and multifaceted pathobiology of oral mucositis (Sonis, 2004b), diverse interventions have been tested (Lalla et al., 2014). Based on these studies, the Multinational Association of Supportive Care in Cancer/National Society of Oral Oncology (MASCC/ISOO) recently revised evidence-based clinical practice guidelines for oral mucositis care (Table 1). In general, oral mucositis care involves basic oral care and control of symptoms through the use of antimicrobials, coating agents, anesthetics, and analgesics. Oral mucositis interventions are focused on growth factors and anti-inflammatory agents (Lalla et al., 2014). Although several growth factors, such as KGF (Henke et al., 2011; Le, 2011; Weigelt et al., 2011), epidermal growth factor (EGF) (Epstein et al., 2000; Wu et al., 2009), and granulocyte-colony-stimulating factor (G-CSF) (Jyung et al., 1994; Raber-Durlacher et al., 2013) have been explored for oral mucositis treatment, Palifermin (truncated KGF) is the only US FDA approved drug recommended by MASCC/ISOO for oral mucositis prevention (Spielberger et al., 2004; Raber-Durlacher et al., 2013). The major concern when using a growth factor to treat oral mucositis is the potential oncogenic effect that would compromise cancer treatment. Among studied anti-inflammatory agents, benzydamine is an effective antioxidant, anti-inflammatory agent, and inhibitor of leukocyte-endothelial interactions (Epstein et al., 2001), also having analgesic, anesthetic, and antimicrobial activities (Rubenstein et al., 2004). Therefore, the MASCC/ISOO guidelines recommend benzydamine mouthwash for the prevention of radiation-induced mucositis in patients with head and neck cancer receiving moderate dose radiotherapy (Nicolatou-Galitis et al., 2013). However, not all anti-inflammatory agents are effective at alleviating oral mucositis (Lalla et al., 2014).

**TABLE 1:** MASCC/ISOO clinical practice guidelines for care of patients with oral mucositis

Intervention	Purpose	Treatment category/patient population	Guideline
*Basic oral care*			
Oral care	Prevention	All patients	Suggested
Chlorhexidine mouthwash	Prevention	Radiotherapy for HNC	Suggested
*Cytokines and growth factors*			
Palifermin (truncated KGF)	Prevention	High-dose chemotherapy and total body irradiation followed by autologous stem cell transplantation for a hematological malignancy	Recommended
*Anti-inflammatory agents*			
Benzydamine mouthwash	Prevention	Moderate dose radiation therapy (up to 50 Gy), without concomitant chemotherapy in patients with HNC	Recommended
*Antimicrobials, coating agents, anesthetics, and analgesics*			
Patient-controlled analgesia with morphine	Treat pain	HSCT	Recommended
Transdermal fentanyl	Treat pain	Conventional or high-dose chemotherapy, with or without total body irradiation	Suggested
2% morphine mouthwash	Treat pain	Radiotherapy for HNC	Suggested
0.5% doxepin mouthwash	Treat pain	Radiotherapy for HNC	Suggested
*Device directed therapies*			
Low-level laser (wavelength at 650 nm)	Prevention	HSCT conditioned with high-dose chemotherapy, with or without total body irradiation	Recommended
Low-level laser (wavelength ~632.8 nm)	Prevention	H&N RT without concomitant chemotherapy	Suggested
Cryotherapy	Prevention	Bolus 5-fluorouracil chemotherapy High-dose melphalan, with or without total body irradiation, as conditioning for HSCT	Recommended Suggested
*Natural and miscellaneous agents*			
Zinc supplements	Prevention	Radiotherapy or chemoradiotherapy for oral cancer	Suggested

Abbreviations: Gy, grays; HSCT, hematopoietic stem cell transplantation; MASCC/ISOO, Multinational Association of Supportive Care in Cancer and International Society of Oral Oncology; KGF, Keratinocyte growth factor; head and neck radiation therapy (H&N RT) head and neck cancer (HNC)

In addition to therapeutic agents, laser or other light therapy and cryotherapy have also been explored for oral mucositis prevention. Low-level laser therapy has been shown to have photochemical, photophysical, and photobiological effects on cellular metabolism (Migliorati, 2013). Cryotherapy causes vasoconstriction and decreases blood flow to the oral cavity, reducing exposure of the buccal mucosa to chemotherapy (Sorensen et al., 2008). However, its usefulness is limited to only a few chemotherapeutics with short half-lives (Peterson et al., 2013). Further, a large number of natural agents have been explored for oral mucositis treatment (Lalla et al., 2014). Among them, zinc, an essential trace element for some tissue repair processes with antioxidant effects (Yarom et al., 2013), is the only one suggested by the MASCC/ISOO for oral mucositis prevention (Lalla et al., 2014). Overall, among the MASCC/ISOO recommended/suggested therapies, each primarily targets one of the pathological processes of oral mucositis and thus is only moderately effective. This situation highlights the need for a new therapeutic strategy.

## ROLE OF TGFβ SIGNALING IN INFLAMMATION AND ORAL MUCOSITIS

**Figure 1:**
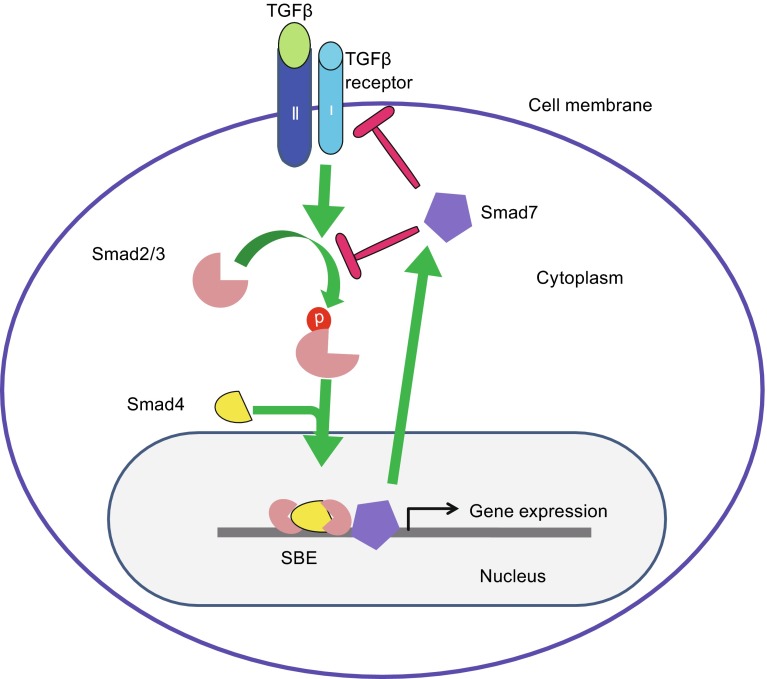
**TGFβ signaling components**. TGFβ signals through type I and type II receptors when TGFβ ligand binds to the type II receptor. The serine/threonine kinase of the type I receptor phosphorylates Smad2 and Smad3, which then bind to Smad4, forming a trimeric complex that translocates from the cytoplasm into the nucleus. The trimeric Smad complex binds to Smad binding elements (SBEs) to regulate gene expression. Smad7 is a nuclear protein. It competes with signaling Smads on SBEs, and translocates to the cytoplasm to block phosphorylation of Smad2/3 or induce degradation of TGFβRI and Smad2/3

TGFβ has three isoforms and TGFβ1 is the predominant form in keratinocytes (Pittelkow et al., 1988). TGFβ signals through type I and type II receptors that have serine/threonine kinase activities and downstream Smad proteins, specifically Smad2 and Smad3 (Fig. 1) (Massague and Gomis, 2006). After ligand binding, the type I receptor phosphorylates Smad2 and Smad3, which then bind to Smad4, forming a trimeric complex that translocates from the cytoplasm into the nucleus. The trimeric Smad complex binds to Smad binding elements (SBEs) to regulate gene expression (Feng and Derynck, 2005; Groneberg et al., 2004).

TGFβ1 was initially identified as an immunosuppressive, anti-inflammatory molecule (Shull et al., 1992; Kulkarni et al., 1993). TGFβ1-deficient mice exhibit inflammation in multiple organs, succumbing to multifocal inflammation and autoimmune disorders in the internal organs (Shull et al., 1992; Kulkarni et al., 1993). However, TGFβ1 also has a pro-inflammatory effect. Levels of TGFβ1 increase rapidly following injury due to secretion by keratinocytes, platelets, and macrophages (Singer and Clark, 1999), and increased levels of TGFβ1 rapidly recruit leukocytes to accumulate at the injury site. Infiltrated leukocytes secrete chemokines and inflammatory cytokines stimulating the inflammatory response (Wang et al., 2006). The pro-inflammatory effect of TGFβ1 is further substantiated in K5.TGFβ1 transgenic mice with latent human TGFβ1 overexpression in the skin and oral mucosa (Li et al., 2004a; Lu et al., 2004), where severe inflammation is observed.

Our recent study identified increased TGFβ protein and signaling activities in both human oral mucositis lesions and radiation-induced oral mucositis in mice (Han et al., 2013). Because TGFβ is a potent epithelial cell growth inhibitor, activated TGFβ induces growth arrest of irradiated epithelial cells. Arresting these cells could allow them to repair damaged DNA, but severe growth arrest also contributes to the thinning of epithelial layers and progression to an ulcer. Additionally, TGFβ induces apoptosis, potentially contributing greatly to the massive cell death found in oral mucositis. Further, our previous study showed that TGFβ1 activates NF-κB (Li et al., 2004b) and elicits severe inflammation in oral mucosa (Lu et al., 2004). Excessive inflammation in oral mucositis could therefore be largely due to TGFβ overexpression. Our study suggests that TGFβ contributes to multiple pathological processes responsible for oral mucositis development. Further, our previous study has shown that excessive and prolonged TGFβ1 expression in keratinocytes resulted in delayed wound healing (Wang et al., 2006). Therefore, increased TGFβ in oral mucositis would also delay its healing.

## SMAD7: A TGFβ SIGNALING ANTAGONIST AND CROSS-TALK MEDIATOR OF MULTIPLE PATHWAYS IN DISEASES

Smad7, a nuclear protein, was initially identified to block TGFβ signaling through translocation to the cytoplasm to block phosphorylation of Smad2/3 (Hayashi et al., 1997; Nakao et al., 1997; Nakao et al.,2002). Later, Smad7 was found to recruit E3 ubiquitin ligases (Smurf1 and Smurf2) to degrade TGFβRI and Smad2/3 (Kavsak et al., 2000; Lin et al., 2000; Ebisawa et al., 2001). Smad7 also binds to DNA through its MH2 domain to regulate transcription (Yan and Chen, 2011).

Because TGFβ is a potent growth inhibitor for epithelial cells and induces apoptosis in many cell types, Smad7 promotes cell proliferation and survival once it reaches a level sufficient to block TGFβ signaling (He et al., 2002; Han et al., 2011). These functions of Smad7 contribute greatly to its role in promoting wound healing after injuries, when cell proliferation is required (Mallawaarachchi et al., 2005; Saika et al., 2005; Han et al., 2011). Additionally, Smad7 promotes cell migration, required for normal wound healing, through interactions with other pathways. Specifically, Smad7 promotes the MAPK3-p38 complex formation to activate JNK/p38 (Edlund et al., 2003), activates Erk (Han et al., 2011), and recruits adenomatous polyposis coli (APC) to the microtubule (Ekman et al., 2012).

Smad7 is expressed at exceedingly low levels in normal keratinocytes (He et al., 2001), but is often overexpressed under pathological conditions (He et al., 2002; Han et al., 2011). Inflammatory diseases frequently show elevated TGFβ-induced levels of Smad7 expression. Because TGFβ in the skin and oral mucosa primarily functions as a pro-inflammatory cytokine, the anti-TGFβ effect of Smad7 makes it act as an anti-inflammatory molecule. Further, Smad7 directly antagonizes NF-κB, the major inflammation pathway. Smad7 overexpression up-regulates IκBα expression, an NF-κB inhibitor (Wang et al., 2005). Smad7 also disrupts the formation of the protein complex TRAF2/TAK1/TAB2/TAB3 (TNF-receptor associated factor 2/TGFβ-activated kinase 1/TAK-binding protein 2/3), which is essential for activation of the inflammation cascade (Hong et al., 2007). Therefore, the anti-TGFβ and anti-NF-κB activities of Smad7 make it one of the most potent anti-inflammatory molecules in stratified epithelial tissues. Similarly, Smad7 knockout mice developed kidney inflammation (Chung et al., 2009; Chen et al., 2011). In contrast, because TGFβ is a potent immune suppressant in the gut, high levels of Smad7 expression in the gut cause inflammatory bowel disease (IBD) due to the loss of TGFβ responsiveness (Monteleone et al., 2004).

With respect to the role of Smad7 in tumorigenesis, because TGFβ is a potent growth inhibitor and apoptosis inducer, the anti-TGFβ effect of Smad7 is suspected to promote tumor growth and survival. In support of this notion, Smad7 overexpression is associated with poor prognosis in colorectal cancer (Boulay et al., 2001). In experimental models, overexpression of Smad7 can promote malignant transformation and tumorigenicity in the pancreas, gastric, lung, skin, and colon (Kim et al., 2004; Kleeff et al., 1999; Liu et al., 2003; Halder et al., 2005; Luo et al., 2010). In contrast, Smad7 has been shown to inhibit cancer progression and metastasis in many tumor types in experimental models, including melanoma, breast cancer colorectal cancer, head and neck and liver cancers *in vitro* and *in vivo* (Azuma et al., 2005; Javelaud et al., 2005; Leivonen et al., 2006; Rizzo et al., 2011; Wang et al., 2013). Smad7’s anti-inflammatory effect in certain tissues and induction of apoptosis in certain cancer types (Wang et al., 2013) could contribute to its tumor-suppressive effect. Additionally, because TGFβ induces formation of cancer-associated fibroblasts (CAFs) to promote cancer progression, it is not surprising that Smad7 can block this process (Li et al., 2013). Interestingly, a study demonstrated that while Smad7 overexpression in T cells increases colitis severity, it decreases colitis-associated cancer (Rizzo et al., 2011). Therefore, the enhanced immune response could also attack tumor cells, a principle for immunotherapy in cancer.

The tissue/context-specific effects of Smad7 partially explain the dual roles of Smad7 in cancer. Another explanation could be dose-dependent effects of Smad7. For instance, while high levels of Smad7 overexpression driven by adenoviral transduction cause skin tumor progression in immune compromised recipient mice (Liu et al., 2003), we have not observed spontaneous tumor formation in K5. Smad7 transgenic mice that express Smad7 at a modest level, even though this level of Smad7 is sufficient to block Wnt signaling (Han, et al., 2006).

## SMAD7: EFFECTS ON PREVENTION AND TREATMENT OF ORAL MUCOSITIS

We utilized K5.Smad7 mice that express a Smad7 transgene in oral epithelia under the control of a keratin 5 promoter, as an animal model, to study their susceptibility to radiation-induced oral mucositis. We found that these mice are remarkably resistant to radiotherapy-induced oral mucositis. This prompted us to seek a pharmacological approach to deliver Smad7 protein into the oral cavity. Exogenous Smad7 has to be delivered inside cells to its natural cellular location, the nucleus. To this end, we developed a recombinant human Smad7 protein with an N-terminal Tat tag (Tat-Smad7), permitting the proteins to rapidly permeate the cell membrane and enter into the nucleus (Han et al., 2013). To test the efficacy of Tat-Smad7 in oral mucositis prevention, we exposed mice to fractionated cranial radiation and topically applied Tat-Smad7 to the oral cavity daily starting 24 h before irradiation through day 8 after initiation of irradiation and observed the treated tissues on day 9. We found Tat-Smad7 treatment significantlly reduced ulcer incidence and sizes. Next, we tested if Tat-Smad7 can be used to treat existing oral mucositis. We began topical Tat-Smad7 application 6 days after irradiation, (when mucosal damage was obvious) applying daily to day 9 and observed the treated tissues on day 10. Tat-Smad7 treated mice show accelerated healing compared to the control group. Our studies show that the preventive and therapeutic effects of Smad7 are due to blocking multiple pathological processes of oral mucositis as discussed below.

### Anti-inflammatory effects of Smad7

We found that both K5.Smad7 mice and Tat-Smad7 treated mouse oral mucosa have less inflammation than control mice during radiation-induced oral mucositis. In these tissues, pSmad2, a surrogate marker for TGFβ activation, and nuclear NF-κB p50, a surrogate marker for NF-κB activation, were both reduced by Smad7 (Han et al., 2013). Because excessive inflammation and upregulation of pro-inflammatory cytokines are early stage insults, the antagonistic effect of Smad7 on both TGFβ and NF-κB signaling substantially contributes to reduced mucositis formation when Smad7 is used as a therapeutic. Because inflammation further exacerbates radiation-induced tissue damage (Sonis, 2004b), reducing inflammation in pre-exisiting lesions provides a beneficial microenvironment for oral mucositis healing. These data indicate Smad7 is a more efficient anti-inflammatory molecule than other pathway inhibitors that target only the NF-κB pathway or the TGFβ pathway (Han et al., 2011; Han et al., 2013).

### Role of Smad7 in proliferation and apoptosis of epithelial cells

In previous studies, we found that Smad7 increases proliferation but reduces apoptosis in stratified epithelia (He et al., 2002). Overexpression of Smad7 can accelerate epithelial cell proliferation and decrease levels of apoptosis, promoting wound healing by blocking TGFβ induced growth inhibition and apoptosis (Han et al., 2011; Han et al., 2013). In oral mucositis, epithelial proliferation is nearly halted due to the presumed combination of TGFβ-mediated growth inhibition and DNA damage-associated cell cycle arrest after radiation. These are primary targets for the therapeutic effect of Palifermin, a potent growth factor. We found that the proliferative effect of Smad7 in oral mucositis was not as pronounced as that of Palifermin, but is sufficient to restore proliferation to a normal baseline level. Additionally, because Palifermin is given systemically via i.v. injection, all keratinocytes in the body go through hyperproliferation in response to Palifermin treatment. In contrast, Tat-Smad7 can be administered locally, avoiding stimulation of non-target cells, particularly cancer cells away from the ulcer. Because Palifermin does not fully protect cells from damage and apoptosis, hyperproliferation is needed to compensate for lost epithelial cells to prevent oral mucositis. This could explain why Palifermin needs to be administered three days before radiotherapy. In contrast, a recent study has shown that Smad7 enhances DNA repair after radiation by direct interactions with the ATM DNA repair complex (Park et al., 2014). This effect, together with its potent anti-inflammatory effect, could explain why Tat-Smad7 treated oral mucosa has fewer damaged and apoptotic cells than Palifermin-treated mucosa. Reduced DNA damage and cell death would diminish the need for hyperproliferation.

### Role of Smad7 in acceleration of epithelial migration

When using Tat-Smad7 as a therapeutic agent for existing oral mucositis, its role in epithelial migration would be essential to initiate healing. During wound closure, re-epithelialization relies primarily on rapid migration of keratinocytes at the wound edge and proliferation of the basal layer of epithelial cells located adjacent to the wound site (Castilho et al., 2010; Lawson and Burridge, 2014). We found that knocking down Smad7 in normal human oral keratinocytes abrogates cell migration (Han et al., 2013). This phenomenon is strikingly comparable with loss of Rac1, a member of the Rho family of GTPases necessary for oral wound healing and keratinocyte migration (Liu et al., 2009; Castilho et al., 2010). We found that Smad7 increases expression levels and activities of Rac1 (Han et al., 2013). This finding was initially surprising because TGFβ signaling is known to activate Rac1 at the protein level via a Smad-independent mechanism (Derynck and Zhang, 2003). Further analyses revealed that Rac1 is repressed by Smad2/3/4, the protein complex that recruits the transcriptional co-repressor CtBP1. This Smad-mediated Rac1 repression is abrogated when Smad7 prevents binding of Smad2/3/4 and CtBP1 to the SBE site on the Rac1 promoter. Although it remains to be determined if elevated TGFβ is able to activate Rac1 via a non-Smad pathway in the context of oral mucositis, Smad-dependent Rac1 repression could overcome Smad-independent Rac1 activation. When this repression is nullified by Smad7, Rac1-mediated epithelial migration and proliferation are enabled. We also found much higher levels of endogenous Rac1 in oral cancer cells than in normal keratinocytes. In cancer cells, signaling Smads are lost or inactivated so their repression of Rac1 would be absent or other oncogenic mechanisms could independently activate Rac1. This potentially explains why Smad7 preferentially activates Rac1 and promotes migration in normal, but not cancer, epithelial cells.

## SUMMARY AND PERSPECTIVES

**Figure 2:**
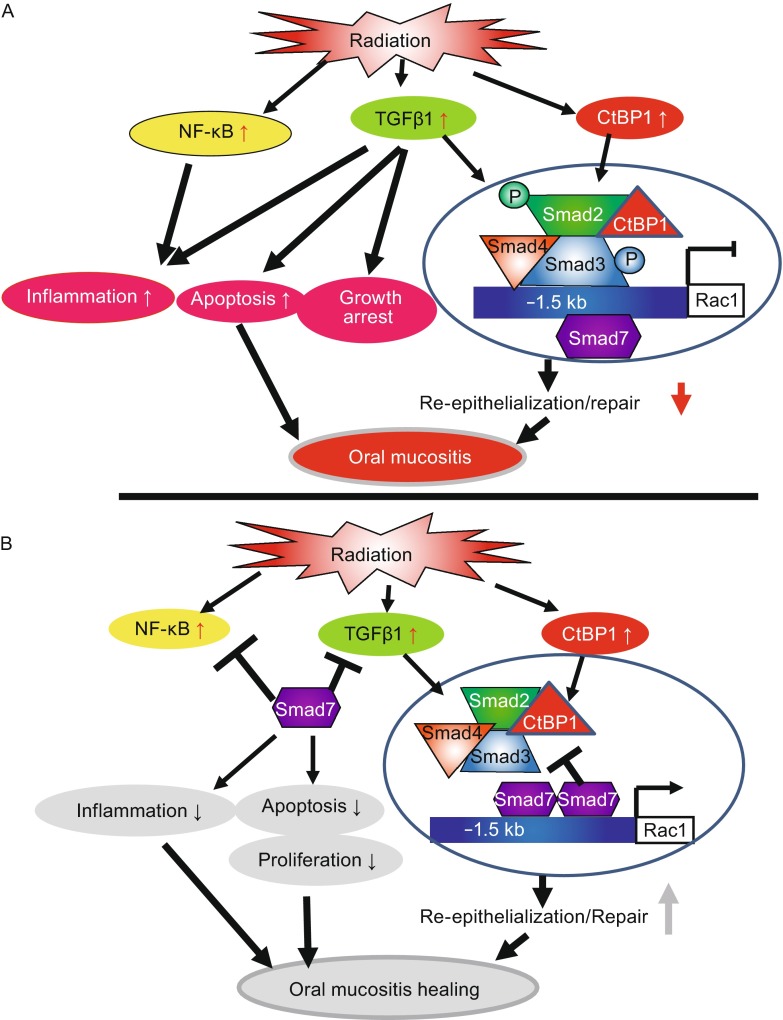
**Summary of potential mechanisms of Smad7-mediated protection and healing of oral mucositis (from Han et al., Nature Medicine, 2013)**. (A) Radiation activates NF-κB, increases TGFβ1 and CtBP1. NF-κB and TGFβ1 induce inflammation. TGFβ1 induces apoptosis, growth arrest, and activates Smads which recruit CtBP1 to the Rac1 promoter to repress Rac1 transcription, leading to blunted re-epithelialization. (B) Smad7 blocks NF-κB and TGFβ1-induced inflammation and blocks TGFβ1-induced apoptosis and growth arrest. Smad7 activates Rac1 by either preventing TGFβ1-mediated Smad phosphorylation or competing with signaling Smads/CtBP1 transcriptional repression complex in the Rac1 promoter. Increased Rac1 induced by Smad7 contributes to keratinocyte migration during re-epithelialization

Oral mucositis has multiple pathological processes, hence targeting one of them is insufficient for therapeutic efficacy. We found that Smad7 has both prophylactic and therapeutic effects on radiation-induced oral mucositis in mice. At the pathological level, Smad7 targets multiple pathological processes required for oral mucositis development, particularly through its effects on proliferation, apoptosis, migration in keratinocytes, and reducing inflammation in the stroma. At the molecular level, Smad7 functions in both the cytoplasm and nucleus to dampen TGFβ and NF-κB pathways and regulates targets to encourage oral mucositis healing (Fig. 2).

Considering the pleotropic effects of Smad7, future challenges to using Smad7-based therapy will be to promote wound healing without blocking TGFβ-mediated tumor-suppressive effects. In most cancer types, loss of TGFβ-mediated tumor suppression occurs at the molecular level for TGFβ signaling components or through other oncogenic pathways that override TGFβ-induced tumor suppressive effects. In these tumors, Smad7 may not be able to affect cancer cell behavior but instead selectively affect healthy mucosa. However, TGFβ signaling is often activated in tumor stroma. Consequently, Smad7 provides tumor suppressive effects through the inhibition of TGFβ- and NF-κB- mediated tumor promotion in tumor stroma (Azuma et al., 2005; Hong et al., 2007; DiVito et al., 2010). The effect of Smad7 on the stroma is consistent with the rationale for using TGFβ inhibitors to treat advanced metastatic cancers in clinical trials. Nevertheless, the dosage and time course need to be carefully assessed to minimize the risk of Smad7 treatment causing a resurgence of cancer cells.

## ABBREVIATIONS

IBD: inflammatory bowel disease
IL-1β: interleukin-1β
IL-6: interleukin-6
KGF: keratinocyte growth factor
MMPs: matrix metalloproteinases
NF-κB: nuclear factor-κB
ROS: reactive oxygen species
TGFβ: transforming growth factor β
TNF-α: tumor-necrosis factor-α
